# Visionary Nursing Leadership: Expanding Nurse Practitioner Roles to Enhance Quality Health Care

**DOI:** 10.7759/cureus.77009

**Published:** 2025-01-06

**Authors:** N. Leena Madhura, K.P. Joshi, Deepak C Jamadar

**Affiliations:** 1 Obstetric and Gynecological Nursing, Sri Venkata Sai (SVS) College of Nursing, Mahabubnagar, IND; 2 Community Medicine, Sri Venkata Sai (SVS) Medical College and Hospital, Mahabubnagar, IND; 3 Biostatistics, Sri Venkata Sai (SVS) Medical College and Hospital, Mahabubnagar, IND

**Keywords:** healthcare access, india, nurse practitioner, nursing leadership, role expansion

## Abstract

Objective

This study aims to identify, determine, and assess the factors influencing nursing leadership and evaluate the effectiveness of interventions to enhance leadership among nurse practitioners (NPs).

Methods

This study employed an exploratory, descriptive qualitative design to examine nurse leaders’ perceptions of nurse practitioner (NP) role expansion in India. Sixty senior nurse administrators were purposively sampled from healthcare and educational institutions in Telangana. Data collection, conducted between May and October 2024, utilized a structured questionnaire divided into quantitative and qualitative sections. Key themes identified through manual thematic analysis included the influence of nursing leadership, perceived benefits of NP roles, barriers to role expansion, and strategies to overcome challenges. Quantitative data, including demographic variables, were analyzed using SPSS software with descriptive statistics, chi-square tests, and ANOVA tests. A pilot study in Karnataka refined the questionnaire, ensuring its clarity and validity. Ethical approval was obtained, and participant confidentiality was maintained, enabling a robust exploration of leadership perspectives on NP role expansion.

Results

Approximately 66.7% of respondents perceived nursing leadership as pivotal in advocating for the NP role. Significant differences were observed in perceived benefits scores between males (3.8 ± 0.6) and females (4.2 ± 0.5). Similarly, significant differences were found in support for role expansion scores between males (3.7 ± 0.7) and females (3.8 ± 0.6). These findings indicate that females perceived greater benefits and support for NP role expansion compared to males. Furthermore, educational background and current positions were significantly associated with perceptions of challenges and support for NP role expansion.

Conclusions

The shortage of primary care physicians has highlighted the need to implement NP-led care in the country. The Indian Nursing Council (INC) has introduced an NP program in critical care nursing. Evidence demonstrates that NPs can effectively deliver a wide range of services, including assessment, diagnosis, treatment of minor ailments, curative and rehabilitative care, and referral services, particularly in underserved and deprived communities.

## Introduction

The healthcare sector increasingly acknowledges the critical role of nurse practitioners (NPs) in providing high-quality care and meeting the rising demand for services [1]. With advanced education and clinical training, NPs deliver a wide range of healthcare services, including diagnosing and managing illnesses, prescribing medications, and promoting health education [2]. Their role is particularly significant in primary care, where there is an urgent need for accessible and cost-effective healthcare providers [3]. Nursing leadership is fundamental in shaping and expanding NP roles, as effective leaders advocate for policies that enhance their scope of practice [4]. By fostering the growth of NP roles, nursing leaders can help alleviate the pressures on healthcare systems, improve patient outcomes, and increase the availability of services [5]. The substantial potential of NPs to contribute to healthcare underscores the importance of strategic nursing leadership in addressing challenges and maximizing opportunities [6].

Despite these opportunities, expanding NP roles faces numerous challenges regarding regulatory barriers, limitations on the scope of practice, and varying acceptance levels among healthcare professionals and the public, which can impede progress [7]. Additionally, the perceptions and attitudes of nurse leaders toward NP roles play a critical role, as their support and advocacy are instrumental in overcoming these challenges [8]. Understanding nurse leaders’ perspectives on the benefits, challenges, and strategies of NP role expansion is essential for informed decision-making and policy development.

Therefore, this study was designed to explore nurse leaders’ perceptions of expanding nurse practitioner (NP) roles in India using an exploratory descriptive qualitative approach. The objectives of the study were to identify and describe the roles and responsibilities of nurse practitioners (NPs) within the healthcare system, to explore the influence of nursing leadership in promoting the expansion of NP roles, to assess nurse leaders’ perceptions of the benefits and challenges associated with NP role expansion, and to analyze the impact of demographic variables, such as educational background and professional positions, on their views regarding this expansion. Furthermore, the study sought to develop strategic recommendations for nursing leadership to effectively drive the expansion of NP roles, thereby enhancing their contribution to delivering quality health care.

## Materials and methods

Study type and design

This study employed an exploratory, descriptive qualitative design to examine nurse leaders’ perceptions regarding the expansion of nurse practitioner (NP) roles in India. A qualitative design was considered appropriate as it facilitated an in-depth investigation of participants’ experiences and perceptions, providing a nuanced understanding of the complexities associated with the challenges, benefits, and strategies related to the proposed role expansion. The research focused on key thematic areas, including the influence of nursing leadership, the perceived benefits of expanding NP roles, barriers to their expansion, and strategies for effective implementation.

Sampling technique

A purposive sampling technique was utilized to select participants who possessed specific expertise and substantial professional experience relevant to the research objectives. This non-probability sampling method ensured the inclusion of individuals who could provide informed and credible insights. The sample consisted of sixty nurse administrators from various cadres, including nursing registrars, district public health nurses (DPHNs), nursing superintendents (NSs), deputy nursing superintendents (DNSs), nursing officers, principals, vice-principals, and professors. The sample size was determined based on the principle of data saturation, ensuring no additional themes emerged as data collection progressed.

Study setting

The research was conducted at SVS College of Nursing, Mahabubnagar, Telangana, India. Participants were recruited from a variety of healthcare and educational institutions, including primary health centers (PHCs), hospitals, nursing colleges, and nursing schools. This diversity facilitated the collection of data that reflected a wide range of perspectives and institutional contexts. Data collection was conducted over six months, from May to October 2024.

Inclusion and exclusion criteria

Participants were included in the study if they held senior leadership positions in nursing and demonstrated substantial administrative experience. This inclusion criterion ensured that respondents were capable of providing valuable insights into nursing leadership and NP role expansion. Individuals who declined to participate were excluded to maintain the integrity and engagement of the responses.

Data collection method

The data collection process employed a structured questionnaire, which was distributed to participants via email. This approach allowed respondents to complete the questionnaire at their convenience, enabling the collection of both detailed quantitative data and nuanced qualitative insights. The demographic data collected in the quantitative section formed the basis for statistical analysis, while the qualitative responses facilitated an in-depth exploration of participants’ perceptions and experiences. Data collection for the main study was conducted between August 13 and September 4, 2024, and included participants from multiple states across India, ensuring the representation of diverse perspectives.

Questionnaire

The primary data collection instrument was a structured questionnaire meticulously designed to gather both quantitative and qualitative data (Appendix 1). The questionnaire was divided into two sections to comprehensively address the study’s objectives. The first section consisted of quantitative items aimed at collecting demographic information, including participants’ age, gender, professional designation, years of experience, educational background, and institutional type. These close-ended questions were systematically scored and coded to facilitate statistical analyses. 

The second section of the questionnaire consisted of open-ended questions aimed at gathering qualitative data aligned with the study’s thematic framework. Participants provided in-depth insights into key topics, including the influence of nursing leadership, the perceived benefits of expanding NP roles, the challenges and barriers associated with their implementation, and recommended strategies for overcoming these challenges. The qualitative data were analyzed using thematic analysis, a structured method for identifying, organizing, and interpreting patterns of meaning within the data. The process involved several systematic stages: familiarization with the data, generating initial codes, identifying and categorizing themes, and subsequently reviewing, refining, and defining the final themes. To ensure the reliability of the analysis, multiple researchers independently reviewed the data, and any discrepancies in coding or theme identification were resolved through collaborative discussion. This rigorous approach ensured that the derived themes were both reflective of the data and aligned with the study’s overarching objectives. Qualitative data analysis was conducted manually, as the manageable size of the dataset did not necessitate the use of specialized software. Manual coding allowed for the systematic identification of themes and patterns in alignment with the study objectives. Coding and analysis were carefully documented to maintain transparency and replicability of the findings. Where appropriate, representative quotes from participants were extracted and included in the results section to illustrate the identified themes.

The questionnaire underwent a pilot study conducted in Karnataka State from July 8 to July 14, 2024, to test its clarity, relevance, and effectiveness. Feedback from the pilot participants indicated areas where improvements were needed, including rewording ambiguous questions, clarifying instructions for open-ended questions, and ensuring the demographic questions captured the necessary details. These refinements improved the instrument’s overall usability and ensured it was fully aligned with the objectives of the main study. To further ensure the reliability and validity of the findings, triangulation was employed. This involved comparing and cross-referencing insights from the quantitative and qualitative data to confirm consistency and coherence. Member checking was also utilized for qualitative responses, where participants were given the opportunity to review and confirm the accuracy of the themes derived from their input. This approach enhanced the credibility and trustworthiness of the qualitative findings.

Questionnaire reliability and validation

The reliability and validity of the questionnaire were rigorously evaluated to ensure its appropriateness as a data collection tool. Content validity was established through an extensive review of the relevant literature and consultation with subject matter experts, ensuring that the instrument comprehensively addressed the study objectives. Construct validity was verified by aligning the questionnaire items with established theoretical frameworks in nursing leadership and NP role expansion. Face validity was confirmed by expert reviewers who evaluated the clarity, relevance, and comprehensiveness of the questions. Reliability was assessed using Cronbach’s alpha, yielding a value exceeding 0.70, which indicated strong internal consistency. Additionally, pilot testing with five participants facilitated the identification and resolution of ambiguities, further enhancing the robustness of the questionnaire.

Statistical analysis

Quantitative data were analyzed using IBM SPSS Statistics for Windows, Version 24 (Released 2016; IBM Corp., Armonk, New York, United States). Descriptive statistics, including means, standard deviations, frequencies, and percentages, were used to summarize demographic characteristics and responses. Associations between demographic variables and perceptions related to NP role expansion were examined using chi-square tests, with a significance level set at p < 0.05. Independent sample t-tests were conducted to compare differences in perceptions across participant groups, and one-way ANOVA was employed to explore variations in perceptions based on educational background, followed by post-hoc analyses for significant results. Qualitative data were analyzed thematically, identifying recurring patterns and unique insights into the influence of leadership, perceived benefits, barriers, and recommendations for expanding NP roles.

Ethical considerations

This study adhered to established ethical standards for research involving human participants. Ethical approval was obtained from the Institutional Review Board (IRB) at SVS College of Nursing. Prior to participation, all respondents were informed about the purpose, procedures, potential risks, and benefits of the study, as well as their right to withdraw at any point without penalty. Written informed consent was obtained, and participant confidentiality was safeguarded by anonymizing data and securely storing information. These measures ensured compliance with ethical guidelines and promoted participant trust.

## Results

Table 1 presents the demographic profile of the study participants (N = 60), highlighting a diverse cohort. Age distribution revealed that 25 (41.7%) participants were over 51 years old, 20 (33.3%) were between 41-50 years, and 15 (25%) were aged 30-40 years. The sex distribution showed a predominance of females, 40 (66.7%), compared to males, 20 (33.3%). Regarding educational qualifications, 40 (66.7%) participants held postgraduate degrees or higher, 15 (25%) were graduates, and five (8.3%) held diplomas. Participants occupied various professional positions, with 20 (33.3%) serving as nursing officers, followed by 10 (16.7%) each as principals, professors, and assistant professors, while public health nurses and nursing registrars accounted for five (8.3%) each. Regarding work settings, 30 (50%) participants were affiliated with colleges of nursing, while the remaining 30 participants were evenly distributed across schools of nursing, hospitals, and community settings (10, 16.7% each). Experience in their current roles varied, with 20 (33.3%) participants having over 15 years of experience and 15 (25%) having one to five years. Notably, 40 (66.7%) participants had over 15 years of overall nursing experience, underscoring a highly experienced cohort that provided valuable insights for the study.

**Table 1:** Demographic information of participants (N = 60) DPHN: district public health nurses

Characteristic	Category	Total N (%)
Age	30-40	15 (25%)
	41-50	20 (33.3%)
	>51	25 (41.7%)
Gender	Male	32 (53.3%)
	Female	28 (46.7%)
Educational Background	Diploma	5 (8.3%)
	Graduation	15 (25%)
	Post-Graduation & Above	40 (66.7%)
Present Position/Designation	Principals	10 (16.7%)
	Professor	10 (16.7%)
	Assistant Professor	10 (16.7%)
	DPHN	5 (8.3%)
	Nursing Registrars	5 (8.3%)
	Nursing Superintendents	10 (16.7%)
	Nursing Officers	10 (16.7%)
Working Area	School of Nursing	10 (16.7%)
	College of Nursing	30 (50%)
	Hospital	10 (16.7%)
	Community	10 (16.7%)
Years of Experience in Current Position	1-5 years	15 (25%)
	6-9 years	10 (16.7%)
	10-15 years	15 (25%)
	>15 years	20 (33.3%)
Years of Experience in Nursing	1-5 years	5 (8.3%)
	6-9 years	5 (8.3%)
	10-15 years	10 (16.7%)
	>15 years	40 (66.7%)

Table 2 summarizes participants’ responses regarding the influence of nursing leadership on nurse practitioner (NP) role expansion. Participants identified several key strategies through which nursing leadership can support NP roles, including encouraging NP adoption, setting protocols, collaborating with government and regulatory bodies, and advocating for NP roles. Specific strategies already implemented or observed by participants included issuing government orders, fostering interdisciplinary collaboration, mentoring, advocating for NP roles, and establishing training centers. The reported benefits of NP role expansion included improved healthcare accessibility, reduced healthcare costs, decreased workload for physicians, and an enhanced professional image of nurses.

**Table 2:** Responses on influence of nursing leadership NP: nurse practitioner

Question	Summary of Responses
How can nursing leadership support the expansion of NP roles?	Encouraging NP roles, setting protocols, collaborating with government and regulatory bodies, advocacy.
What strategies have you implemented or witnessed to support NP role expansion?	Government orders, interdisciplinary collaboration, mentoring, advocacy, establishing training centers.
What are the potential benefits of expanding NP roles in quality healthcare?	Improved accessibility, reduced healthcare costs, workload reduction for doctors, enhanced professional image.

Table 3 outlines the challenges identified by participants in expanding NP roles. Key barriers included non-acceptance by the medical fraternity, the absence of a legal framework, professional conflicts, and limited career progression opportunities. Participants suggested actionable measures to address these challenges, such as enacting a dedicated NP Act, developing comprehensive policies, conducting awareness programs, and establishing clear job descriptions. Specific barriers encountered or anticipated included resistance from medical practitioners, concerns over patient safety, and the lack of defined career pathways or cadres for NPs.

**Table 3:** Challenges to expanding nurse practitioner roles NP: nurse practitioner

Question	Summary of Responses
What challenges do you foresee in expanding NP roles?	Non-acceptance by medical fraternity, lack of legal framework, conflicts, lack of career progression.
How can these challenges be addressed effectively?	Enacting NP Act, developing policies, awareness programs, establishing clear job descriptions.
Specific barriers encountered or anticipated in NP role expansion?	Resistance from medical practitioners, safety concerns, and no cadre or career ladder.

Table 4 provides participants’ recommendations for nursing leaders to facilitate NP role expansion. Proposed strategies included lobbying the government, drafting policy documents, creating cadre posts, and conducting awareness seminars to promote the importance of NP roles within the healthcare system.

**Table 4:** Recommendations and strategies for NP role expansion NP: nurse practitioner

Question	Summary of Responses
What strategies would you recommend for nursing leaders to drive NP role expansion?	Lobbying with government, developing policy documents, creating cadre posts, conducting awareness seminars.

Table 5 reports the results of independent samples t-tests to evaluate gender differences in perceptions of NP role expansion. A significant difference was found in perceived benefit scores, with females (mean = 4.2 ± 0.5) scoring higher than males (mean = 3.8 ± 0.6), resulting in a mean difference of -0.4 (t = -2.12, p = 0.038). However, no significant gender difference was observed in support for role expansion scores (females: mean = 3.8 ± 0.6; males: mean = 3.7 ± 0.7), with a mean difference of -0.1 (p = 0.235). These findings suggest that females perceived more significant benefits of NP role expansion than males, though overall support for NP roles did not differ significantly by gender.

**Table 5:** Independent samples t-test results

Variable	Group 1 (Male)	Group 2 (Female)	Mean Difference	t-value	p-value	Interpretation
Perceived Benefits Score	3.8 ± 0.6	4.2 ± 0.5	-0.4	-2.12	0.038	Significant difference
Support for Role Expansion Score	3.7 ± 0.7	3.8 ± 0.6	-0.1	-1.20	0.235	No significant difference

Table 6 presents the results of the chi-square test examining associations between variables. A significant relationship was found between educational background and perceived challenges in NP role expansion (χ² = 10.42, df = 3, p = 0.015). Similarly, participants’ professional positions were significantly associated with their support for NP role expansion (χ² = 8.73, df = 2, p = 0.013). These results highlight that participants’ educational qualifications and professional roles influenced their perceptions of challenges and support for NP role expansion.

**Table 6:** Association between demographic variables and perceptions of NP role expansion NP: nurse practitioner

Variable	Chi-square (χ²)	Degrees of Freedom (df)	p-value	Interpretation
Educational Background vs. Perceived Challenges	10.42	3	0.015	Significant association
Position vs. Support for Role Expansion	8.73	2	0.013	Significant association

Table 7 summarizes the results of a one-way ANOVA conducted to explore the relationship between years of nursing experience and perceived challenges in NP role expansion. The ANOVA revealed a statistically significant difference between experience groups (F = 3.42, p = 0.022). The between-group sum of squares (SS) was 4.76 (df = 3, MS = 1.59), while the within-group SS was 26.44 (df = 56, MS = 0.47). These findings suggest that years of nursing experience significantly influence perceptions of the challenges associated with NP role expansion.

**Table 7:** One-way ANOVA test results for experience levels and perceived challenges in nurse practitioner role expansion

Source of Variation	Sum of Squares (SS)	Degrees of Freedom (df)	Mean Square (MS)	F-value	p-value	Interpretation
Between Groups (Experience Levels)	4.76	3	1.59	3.42	0.022	Significant difference
Within Groups	26.44	56	0.47			
Total	31.20	59				

## Discussion

The role of nurse practitioners (NPs) in the healthcare system has undergone significant transformation in recent decades due to the increasing demand for healthcare services and the evolving complexity of patient needs. Expanding the scope of NP practice has emerged as a crucial strategy to address healthcare workforce shortages, particularly in underserved areas while enhancing the quality of care [9]. This study highlighted the critical influence of nursing leadership in facilitating NP role expansion. Participants emphasized that effective leadership is essential for advocating policy changes, providing ongoing education and training, and fostering a supportive environment for NPs. This aligns with existing literature, which suggests that nursing leaders shape organizational cultures and policies, enabling NPs to practice fully [10]. Strategies such as mentorship programs, advocacy for an expanded scope of practice, and interprofessional collaboration were identified as effective methods to support NP role expansion. Previous research supports these findings, indicating that strong nursing leadership correlates with favorable NP practice environments and improved patient outcomes [11,12]. These findings underscore the importance of leadership in promoting NP role expansion, particularly in contexts where traditional hierarchies may limit the potential of NPs.

Expanding NP roles offers substantial benefits to healthcare systems. Participants highlighted that NPs improve access to care in rural and underserved areas, addressing critical gaps in healthcare delivery caused by physician shortages. In urban areas, NPs contribute by enhancing healthcare accessibility in densely populated regions, reducing patient wait times, and improving continuity of care. Their advanced training enables them to manage a wide range of conditions, which alleviates the burden on overextended healthcare facilities in city settings. This dual impact ensures that both rural and urban populations benefit from more efficient and accessible healthcare services. By providing primary and specialized care, NPs can reduce wait times and enhance patient satisfaction [13]. Additionally, expanding NP roles has been linked to better patient outcomes, with NPs employing a holistic approach that prioritizes health promotion, disease prevention, and patient education [14]. Participants also emphasized the cost-effectiveness of employing NPs, as they can lower healthcare costs by reducing unnecessary hospital admissions and emergency visits, consistent with previous research findings [15]. Furthermore, NPs address healthcare disparities in underserved communities, as they are often more willing to work in these areas than physicians [16]. Studies, such as Auerbach et al., have shown that NP-provided care significantly improves health outcomes in marginalized populations by increasing access to essential services [17].

Despite these benefits, several challenges persist in expanding NP roles. Participants identified restrictive scope-of-practice laws as significant barriers, limiting NPs’ independence, prescribing abilities, and procedural authority [18]. These regulatory barriers are associated with lower job satisfaction and difficulty providing comprehensive care, particularly in states with more restrictive environments [19]. Acceptance among healthcare professionals, especially physicians, also poses a challenge, often stemming from concerns about care quality and professional competition. Additionally, a lack of public awareness regarding NP roles contributes to underutilization, with patients usually reluctant to seek NP care due to misconceptions [20]. Addressing these challenges requires educating the public about NP qualifications and expertise. Research shows informed patients are more likely to accept NP-provided care and report higher satisfaction [21].

The analysis of demographic variables revealed that age, years of experience, and educational background influenced participants’ perceptions of NP role expansion. Older participants were more likely to support NP role expansion, reflecting an understanding of the healthcare system’s needs and NPs’ potential contributions. This finding aligns with observations that experienced professionals are more open to innovations that enhance patient care [1]. Similarly, participants with higher education levels, particularly those with doctoral degrees, demonstrated more substantial support for NP role expansion, likely due to their more profound understanding of healthcare complexities. However, the lack of significant differences between participants with bachelor’s and master’s degrees suggests that support for NP role expansion may already be fostered at the undergraduate level. This indicates that targeted educational interventions at this stage could maximize support for NP roles [22].

Several strategies were recommended to facilitate the successful implementation of expanded NP roles. Participants emphasized the need to address regulatory barriers through policy advocacy, with nursing leaders and professional organizations working collaboratively to advocate for changes to scope-of-practice laws, enabling NPs to utilize their entire education and training [23]. Fostering interprofessional collaboration was also deemed crucial to overcoming resistance from other healthcare professionals. Interprofessional education and team-based care were highlighted as strategies to promote mutual respect between NPs and physicians, enhancing the effectiveness of healthcare teams [24]. Increasing public awareness of NP roles through education campaigns and community outreach was also recommended to dispel misconceptions and highlight NPs’ contributions to healthcare. Research supports that informed patients are more likely to seek NP services and report higher satisfaction with their care [25].

Limitations of the study

This study has several limitations. The purposive sampling method may limit the generalizability of the findings, as the sample may not fully represent the diversity of perspectives across India’s healthcare system. The reliance on self-reported data introduces the potential for response bias, and the cross-sectional design captures perceptions at a single point in time, lacking insights into changes over time. While the sample size was sufficient for qualitative analysis, it may not adequately reflect the broader population of healthcare professionals. Furthermore, the study focused on nurse administrators, excluding input from frontline nurses and other stakeholders. Future research should consider more extensive, more diverse samples and adopt longitudinal designs to explore changes in perceptions over time and address these gaps.

## Conclusions

This study underscores the need to expand nurse practitioner (NP) roles in healthcare to improve access, enhance patient outcomes, and address existing disparities. While regulatory barriers and limited professional acceptance persist, targeted strategies, including policy advocacy, interprofessional collaboration, public education, and robust nursing leadership offer viable solutions to overcome these obstacles. The findings from this study provide valuable insights that can inform future efforts to integrate NPs into expanded roles more effectively, thereby maximizing their contributions to healthcare delivery and addressing critical system needs.

## Figures and Tables

**Figure 1:**
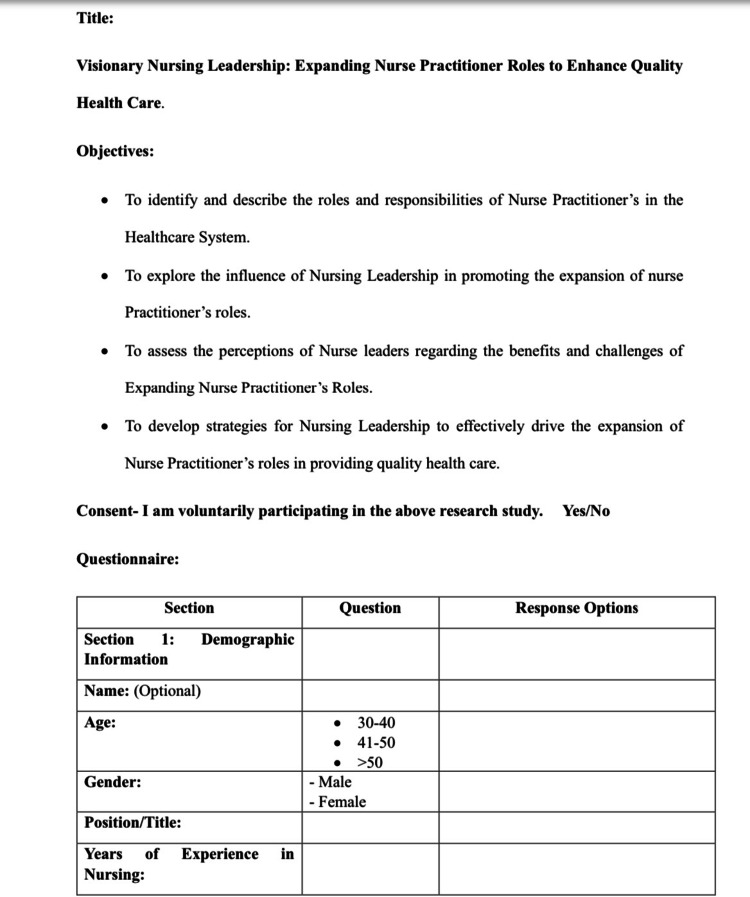
Questionnaire

**Figure 2:**
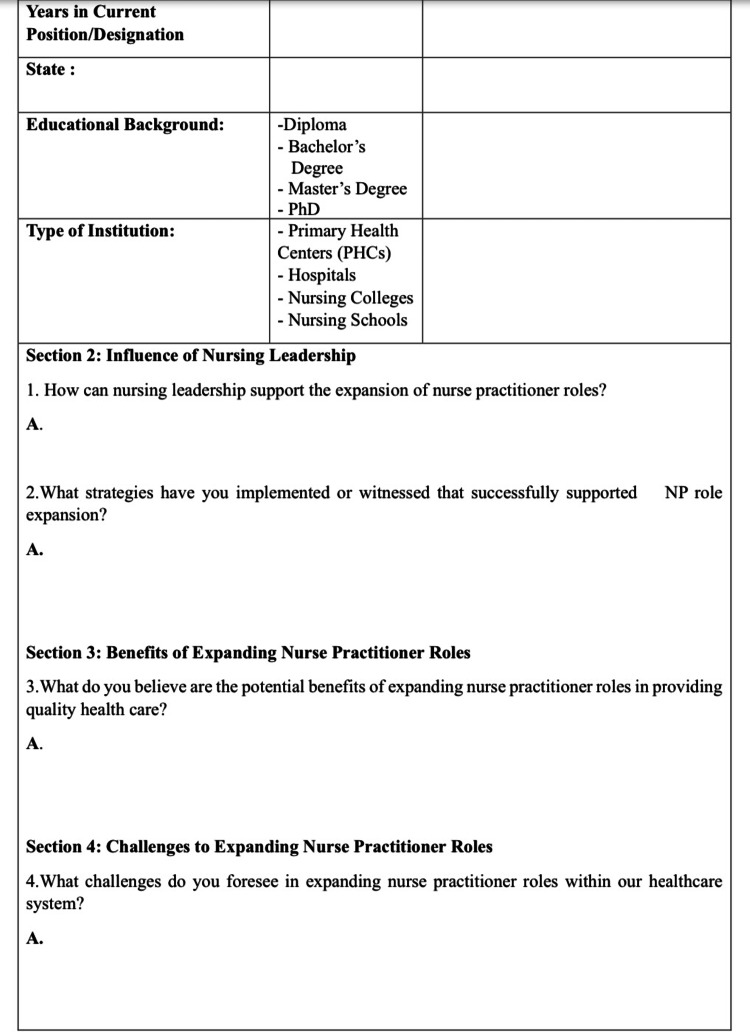
Questionnaire

**Figure 3:**
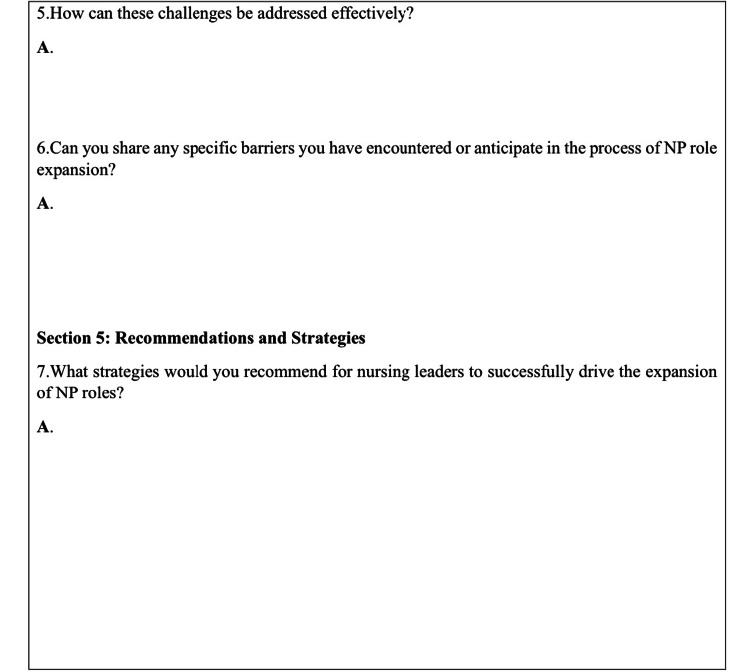
Questionnaire

## Appendices

Appendix 1
